# Towards the Prevention of Freezing of Gait in Parkinson's Disease

**DOI:** 10.1111/ejn.70636

**Published:** 2026-08-19

**Authors:** Anouk Tosserams, Jorik Nonnekes, Bastiaan R. Bloem, Elbrich M. Postma

**Affiliations:** ^1^ Department of Neurology, Center of Expertise for Parkinson & Movement Disorders, Donders Institute for Brain, Cognition and Behaviour Radboud University Medical Center Nijmegen the Netherlands; ^2^ Department of Rehabilitation, Center of Expertise for Parkinson & Movement Disorders, Donders Institute for Brain, Cognition and Behaviour Radboud University Medical Center Nijmegen the Netherlands; ^3^ Department of Rehabilitation Sint Maartenskliniek Nijmegen the Netherlands

**Keywords:** disease modification, freezing of gait, levodopa, Parkinson's disease, prevention, vascular prophylaxis

## Abstract

Despite optimal management, freezing of gait (FOG) remains a disabling complication of Parkinson's disease (PD), contributing substantially to falls, loss of independence and reduced quality of life. This highlights the pressing need for a shift towards preventive approaches, aiming to postpone or even prevent its development. In this narrative review, we examine potential avenues for the secondary prevention of FOG. We discuss how FOG might be prevented specifically, by targeting modifiable risk factors that seem associated with the development of FOG. Examples of potentially effective strategies here include vascular prophylaxis and perhaps avoidance of pulsatile levodopa administration. Secondary prevention efforts also include generic disease‐modifying strategies, including pleiotropic interventions such as exercise and nutrition, targeting either persons in the prodromal disease phase or persons with clinically manifest PD, who are still free from FOG. We conclude by discussing future avenues towards FOG prevention.

## Graphical abstract

Freezing of gait (FOG) is a disabling complication of Parkinson's disease (PD) that remains difficult to treat. Targeting modifiable risk factors and implementing pleiotropic (lifestyle) interventions before FOG onset may potentially delay or prevent its development.

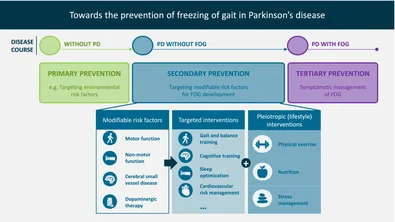

**Abbreviations**
FOG: freezing of gait
MDS‐UPDRS‐III: Movement Disorders Society–Unified Parkinson's Disease Rating Scale Part III
MIND: Mediterranean‐DASH Intervention for Neurodegenerative Delay
PD: Parkinson's disease
SVD: small vessel disease

## 1 Introduction

Freezing of gait (FOG) is one of the most disabling complications of Parkinson's disease (PD) and various other forms of parkinsonism. It is clinically defined as paroxysmal episodes during which the affected person experiences an inability to step effectively, despite attempting to do so (Gilat et al. 2026). The subjective experience of this phenomenon is often described as if the person's feet are being ‘glued to the floor’. As the disease progresses, FOG becomes increasingly prevalent and also refractory to therapeutic interventions, contributing substantially to falls, loss of independence and reduced quality of life (Nutt et al. 2011).

Although considerable progress has been made in the symptomatic management, FOG remains notoriously difficult to treat in a satisfactory way (Tosserams et al. 2025). Pharmacological strategies, including optimization of dopaminergic therapy, may alleviate FOG in some patients. Yet therapeutic responses are often partial, fluctuating or even absent, particularly in later disease stages or in persons with so‐called levodopa‐resistant FOG (McKay et al. 2019; Bloem et al. 2004). Advanced therapies, such as deep brain stimulation, can be beneficial in selected individuals, but only for FOG events that still remained responsive to dopaminergic pharmacotherapy. The effects on FOG are heterogeneous and may diminish over time (Fasano et al. 2015; Zampogna et al. 2022). Notably, FOG has even been described as an adverse late consequence following initiation of DBS (van Nuenen et al. 2008). One of the more effective approaches is offered by nonpharmacological interventions, including the application of a wide range of compensation strategies (Nonnekes et al. 2019). These approaches can effectively reduce the frequency or severity of FOG in controlled settings, although interindividual differences in response are prominent (Tosserams et al. 2022; Cosentino et al. 2023). Moreover, translating these benefits to daily‐life mobility can be challenging, for example, because some find it difficult to consistently apply the right strategy at the right time (Tosserams et al. 2021, 2022). Finding an optimal, personally tailored strategy depends on the availability of skilled professionals (such as physiotherapists, occupational therapists or physiatrists) (Tosserams and Nonnekes 2022), but these are not readily accessible for most persons with PD (Zaman et al. 2021). Finally, there are questions about the long‐term sustainability of effects (Tosserams et al. 2023).

Taken together, these considerations highlight the need to move beyond a purely reactive, symptom‐focused approach. A shift towards a more anticipatory, proactive model of care is warranted: delaying or even preventing the emergence of FOG and mitigating its progression.

## 2 Types of Prevention Strategies

**FIGURE 1:**
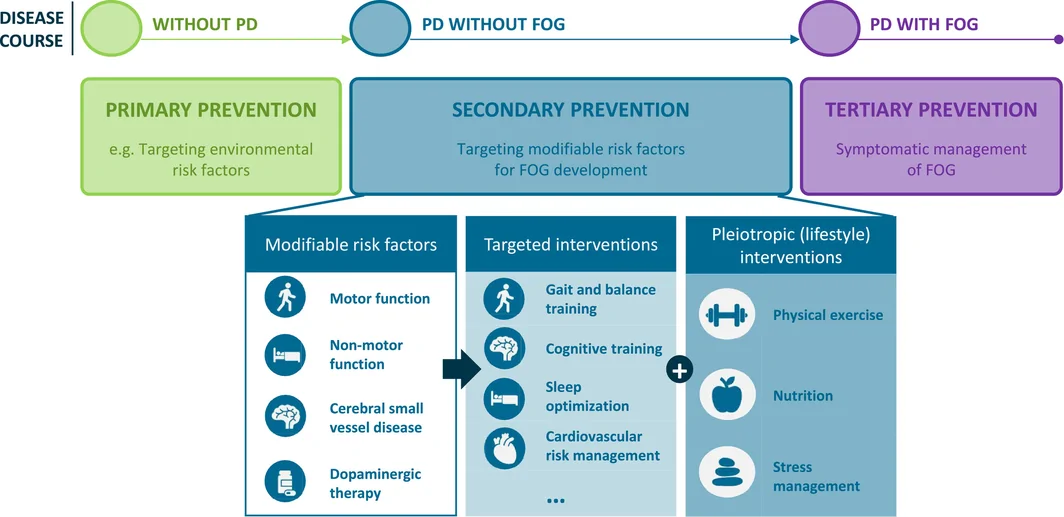
Conceptual framework for freezing of gait prevention across disease stages. FOG, freezing of gait; PD, Parkinson's disease.

Preventive strategies can be conceptualized using the classical public health framework of primary, secondary and tertiary prevention, which distinguishes interventions according to the stage of disease and the timing at which prevention is applied (Figure 1). *Primary prevention* aims to prevent a disease altogether, that is, before any symptoms or underlying pathophysiological processes develop. In the context of FOG, this involves strategies that aim to reduce the incidence of PD at the population level. Examples include reducing exposure to environmental risk factors associated with PD, such as pesticides, air pollution or solvents (Dorsey et al. 2025). Although such approaches are important for reducing the future burden of PD, these fall outside the scope of the present work.

*Secondary prevention* encompasses early identification of, and deploying disease‐modifying interventions in, populations where the underlying pathophysiological processes have started but who have not yet developed FOG. Such secondary prevention strategies can be deployed in two different types of populations. The first group consists of individuals with prodromal parkinsonism and who are at increased risk of developing PD. Some of these persons may still be completely clinically asymptomatic (e.g., identified because of underlying genetic risk that predisposes to future development of PD; Funayama et al. 2023), whereas others may already exhibit early manifestations of what may later evolve into a full‐blown parkinsonian syndrome. One example in the latter category is individuals with isolated REM sleep behaviour disorder, a prodromal condition that is strongly associated with future development of PD or other related synucleinopathies (Iranzo et al. 2006; St Louis et al. 2017). These prodromal populations are theoretically attractive for testing the merits of putative disease‐modifying trials, because there is still much healthy neuronal tissue left that can potentially be rescued from succumbing to the process of neurodegeneration. An example of such a prodromal trial is the SLOW‐SPEED study, which tests the disease‐modifying potential of an exercise‐based intervention (NCT06993142). If proven to be successful, such preventive strategies will delay or even prevent the onset of PD, thereby also indirectly lessening the impact of FOG. This approach also falls outside the scope of the present work.

Our focus here is on the second eligible group for secondary prevention, namely, those individuals who have already developed clinically manifest PD, but who have not yet developed FOG. For this population, the aim is preventing or delaying the onset of FOG and slowing its progression. Specifically, we will review which modifiable risk factors are associated with a higher risk of developing or worsening of FOG. We then highlight potential avenues that, when applied early in the disease process, may help delay or even prevent FOG onset in those individuals who are at increased risk. Note that this review primarily focuses on the prevention of FOG in persons with PD, as experience with putative disease‐modifying strategies in atypical parkinsonian disorders remains limited, and the underlying pathophysiological mechanisms differ from those in PD.

Finally, *tertiary prevention* refers to symptomatic management aimed at reducing the impact and complications of FOG once it has emerged. These approaches have been reviewed comprehensively in a recent publication (Tosserams et al. 2025) and are also addressed in a companion paper in this special issue (Hausdorff et al. 2026).

## 3 Risk Factors Associated With FOG Development and Potential Preventative Strategies

Secondary prevention of FOG starts with timely identification of persons with PD who have the relatively greatest risk of developing FOG. Depending on the population under study, up to 85% of persons with PD may develop FOG (Zhang et al. 2021), so preventive efforts should de facto be focused on almost the entire PD population. However, identification of high‐risk individuals will permit enrichment of trial populations and thereby reduce the required sample size. It may also be interesting to have a more detailed look at the 20% of persons that *never* go on to develop FOG, in an attempt to identify possible *protective* factors.

Many observational studies have evaluated factors associated with FOG incidence. A landmark systematic review and meta‐analysis of 35 observational trials identified multiple factors that were independently associated with the future development of FOG, many of which may already be present years before FOG becomes clinically evident (Herman et al. 2023). Of 8973 people with PD included in this meta‐analysis, 2175 (24.2%) developed FOG by the end of follow‐up (mean: 4.1 ± 2.7 years). This study identified several nonmodifiable independent predictors, including an older age at disease onset, longer disease duration, reduced dopamine transporter uptake and reduced smell. Of much more interest from a therapeutic perspective are the potentially modifiable independent risk factors, which we will discuss in more detail in the following sections.

For each independent risk factor, we will discuss potential preventative strategies. No studies so far have specifically investigated primary or secondary prevention of FOG. In theory, any intervention that slows overall PD progression is also expected to delay the onset or slow the progression of FOG in at‐risk individuals. A combination of targeted interventions that each address specific risk factors for FOG may also yield synergistic effects. Such multimodal strategies have not yet been systematically studied in the context of FOG. To date, most available evidence is derived from relatively small studies evaluating the effects of individual interventions on (other) PD‐related symptoms, rather than on FOG incidence as a primary endpoint. As a result, the potential of combined, targeted approaches to reduce the risk of FOG remains largely unexplored.

### 3.1 Motor Function

Motor features have been associated with an increased risk of developing FOG in PD. For example, greater disease severity, as reflected by higher scores on the Movement Disorders Society Unified Parkinson's Disease Rating Scale part III (MDS‐UPDRS‐III), has consistently been identified as a predictor of incident FOG, independent of disease duration (Giladi, McDermott, Fahn, Przedborski, Jankovic, Stern, and Tanner 2001; Ehgoetz Martens et al. 2018; Herman et al. 2018; Lee et al. 2021; Lo et al. 2019; Ou et al. 2018; Prange et al. 2019; Wang et al. 2022; Xu et al. 2021; D'Cruz et al. 2021; Dadar et al. 2021; Wieler et al. 2016; Kim et al. 2019). In addition, balance and gait impairments are strongly associated with development of FOG. Reduced postural stability, increased gait variability and slower gait speed have all been linked to a higher likelihood of future FOG episodes (Giladi, McDermott, Fahn, Przedborski, Jankovic, Stern, and Tanner 2001; Herman et al. 2018; Lo et al. 2019; Wieler et al. 2016; Forsaa et al. 2015; Sarasso et al. 2022; D'Cruz et al. 2020; Li et al. 2022). These deficits may reflect early dysfunction within neural circuits involved in the control of axial motor function, increasing the vulnerability to FOG.

Targeted interventions focusing on gait adaptability and balance control may strengthen locomotor networks and thereby reduce vulnerability to developing FOG (Tosserams et al. 2025). Although direct prevention of FOG has not been demonstrated, this hypothesis is supported by a meta‐analysis of 41 studies (*n* = 1838 patients) on exercise and training‐based interventions for FOG in PD (Gilat et al. 2021). Training programmes specifically targeting these FOG‐relevant domains yielded larger improvements in self‐reported FOG severity, compared to generic exercise. Modalities included overground adaptive walking programmes, split‐belt treadmill walking, virtual reality–based dual‐task paradigms, repeated platform perturbations and resistance training on unstable surfaces (Putortì et al. 2021; Hulzinga et al. 2023; Bekkers et al. 2020; Silva‐Batista et al. 2020; Ribeiro de Souza et al. 2023). A theoretical framework for selecting the type of training in PD, with the aim of delaying the onset and progression of FOG, has been proposed (Gilat et al. 2021). Although these targeted training programmes seem a promising avenue in the prevention of FOG, the optimal timing of their implementation remains uncertain. Given that FOG ultimately develops in the majority of people with PD (Zhang et al. 2021), there is a compelling rationale for incorporating preventative strategies early in the disease course. However, in the context of limited therapy time and competing rehabilitation priorities, it remains unclear which individuals are most likely to benefit from FOG‐specific interventions and whether these should be prioritized over other therapeutic approaches before gait impairments emerge.

### 3.2 Nonmotor Function

Several nonmotor features of PD have been associated with an increased risk of developing FOG, including anxiety, depression, cognitive dysfunction, sleep disturbances and autonomic dysfunction (Herman et al. 2023). To date, targeted interventions addressing nonmotor dysfunction have largely been evaluated for their ability to improve specific nonmotor symptoms, rather than to prevent the development of FOG. Nevertheless, there is reason to believe that such interventions may also reduce vulnerability to developing FOG. This has been addressed in a comprehensive review on FOG management (Tosserams et al. 2025).

Symptoms of anxiety and depression have consistently been linked to FOG, with a higher affective burden predicting both the presence and future development of FOG, possibly through an impact on attentional resources and limbic‐motor interactions (Ehgoetz Martens et al. 2018; Ou et al. 2018; Wang et al. 2022; Ehgoetz Martens et al. 2017; Banks et al. 2019; Jeong et al. 2021; Zhang et al. 2016). Although pharmacological treatments for anxiety and depression are widely used in clinical practice, evidence specifically addressing their effects on FOG remains limited and inconsistent (Takahashi et al. 2019; Chung et al. 2005).

Cognitive dysfunction, particularly in executive function and attention, is another well‐established risk factor for FOG. Impairments in these domains are thought to compromise the cognitive control of gait, especially under complex or dual‐task conditions, thereby increasing vulnerability to FOG (Ehgoetz Martens et al. 2018; Banks et al. 2019; Zhang et al. 2016; Chung et al. 2021; Kelly et al. 2015). Training paradigms that specifically target cognition‐motor integration, such as dual‐task gait training and enriched, multimodal exercise programmes, have shown promising effects in individuals with FOG. These interventions were associated with improvements in executive function, dual‐task gait performance and, in some studies, reductions in FOG severity (Gilat et al. 2021). Personalized cognitive training programmes targeting executive function have demonstrated modest benefits, including reductions in FOG severity in the ON state, but not the OFF state (Walton et al. 2016; Chow et al. 2021).

Sleep disturbances, including REM sleep behaviour disorder and excessive daytime sleepiness, have also been associated with an increased risk of FOG, potentially reflecting a more widespread neurodegeneration affecting brainstem and hypothalamic circuits involved in arousal and motor control (Wang et al. 2022; Xu et al. 2021; Banks et al. 2019; O'Dowd et al. 2017; Tang et al. 2021). Sleep disturbances are also closely linked to depression and anxiety in PD, and improvement of sleep quality translates into better motor and nonmotor functioning (Minakawa 2022). Sleep disturbances typically require a multimodal treatment approach, which has been comprehensively reviewed elsewhere (Roze et al. 2025; Schütz et al. 2022) and is therefore not discussed in further detail here.

A relatively novel and understudied potential contributor to FOG risk is orthostatic hypotension resulting from autonomic dysfunction, which has been linked to FOG (Herman et al. 2023). Orthostatic hypotension is common in PD and frequently coexists with supine hypertension. This blood pressure instability is clinically relevant in the context of FOG, as both sustained hypertension and supine hypertension are established drivers of cerebral small vessel disease (SVD) (Palma et al. 2020), a putative risk factor for FOG that will be discussed separately below. Indeed, observational studies have suggested that the presence of orthostatic hypotension is associated with a higher burden of SVD in PD (Palma et al. 2020; ten Harmsen et al. 2018). It is therefore reasonable to assume that careful management of orthostatic hypotension—while simultaneously addressing concomitant supine hypertension—could help stabilize 24‐h blood pressure profiles and thereby mitigate progression of SVD in PD, with potentially favourable downstream effects on FOG risk. Importantly, the pharmacological management of orthostatic hypotension and supine hypertension in PD is inherently complex, as interventions targeting one component may exacerbate the other (Fanciulli et al. 2020). A recently published trial has shown the effectiveness of head‐up tilt sleeping in PD on orthostatic drops during the day and supine hypertension at night (van der Stam et al. 2024, 2026).

### 3.3 Cerebral Small Vessel Disease

Cerebral SVD, as reflected by the presence of white matter hyperintensities upon brain imaging, is associated with an increased risk of developing FOG in PD. In a retrospective cohort of 268 individuals with PD, baseline white matter hyperintensities were associated with a higher likelihood of incident FOG over a 5‐year follow‐up period (36% in persons with moderate to severe vs. 14% with mild white matter hyperintensities developed FOG). This association was still present after adjustment for age, sex, dopamine transporter uptake and levodopa equivalent daily dose (Chung et al. 2019).

SVD is commonly observed in individuals with PD and has been linked to worse motor performance—particularly axial impairment—as well as to cognitive decline and mood disturbances, suggesting a contributory role in the pathophysiology of FOG (ten Harmsen et al. 2018; Nanhoe‐Mahabier et al. 2009; Jacob et al. 2023; Bergkamp et al. 2024). Notably, SVD is largely driven by modifiable cardiovascular risk factors, including hypertension, hyperlipidemia, type 2 diabetes and obesity. Indeed, comorbid metabolic syndrome has been associated with a faster deterioration in motor symptoms of PD (Leehey et al. 2017). Moreover, observational studies have linked type 2 diabetes with development and faster progression of PD (Komici et al. 2021). In turn, PD may increase the risk of developing SVD through immobility and related lifestyle changes. Furthermore, levodopa use may elevate serum homocysteine levels, a well‐established risk factor for cardiovascular disorders (Rogers et al. 2003).

These observations could have therapeutic implications. Specifically, in conjunction with blood pressure management through exercise, stress management and dietary interventions, adequate control of other established vascular risk factors contributing to cerebral SVD (e.g., hyperlipidemia, diabetes mellitus and smoking) may help mitigate the risk of FOG. These measures are typically part of standard cardiovascular risk management as implemented in primary care settings. Irrespective of any potential impact on FOG risk, they are essential for improving overall health in individuals with PD.

### 3.4 Dopaminergic Therapy

Levodopa use, higher levodopa equivalent daily doses and the use of catechol‐O‐methyltransferase inhibitors have all been associated with an increased risk of FOG, independent of disease severity (Herman et al. 2023). This complex relationship has been described as the ‘levodopa paradox’, whereby dopaminergic therapy may both alleviate FOG once present, but also contributes to its initial development (Nonnekes et al. 2020). Historically, early clinical descriptions of PD prior to the widespread use of levodopa rarely reported phenomena consistent with FOG, suggesting that its emergence as a prominent clinical feature may, at least in part, be linked to modern treatment paradigms (Koehler et al. 2021). Furthermore, patients with MPTP‐induced parkinsonism showed that severe parkinsonism can exist in the absence of FOG, with freezing phenomena emerging only after initiation of dopaminergic therapy in the severely affected individuals (Nonnekes et al. 2018). More recently, FOG was found to be more commonly present in a cohort of Dutch patients who were treated with levodopa, compared to a cohort of treatment‐naïve patients in Brazil—where access to levodopa is limited (Jansen et al. 2023).

One proposed mechanism is that pulsatile dopaminergic stimulation induces maladaptive plasticity within basal ganglia and cortical networks, thereby contributing to FOG vulnerability (Nonnekes et al. 2020). However, a prospective analysis comparing early start of immediate‐ versus controlled‐release formulations of levodopa in 973 persons within the Oxford Parkinson's disease centre cohort (Jansen et al. 2023) did not demonstrate a reduced risk of FOG with controlled‐release (i.e., less pulsatile) dopaminergic therapy (Ben‐Shlomo et al., unpublished observations).

Current evidence does not support withholding adequately titrated levodopa therapy—particularly in patients with substantial functional disability—in an attempt to reduce the risk of FOG. Accordingly, the still persisting ‘levodopa phobia’ should not be exacerbated in clinical practice (Kurlan 2005). Similarly, the evidence regarding the detrimental effect of catechol‐O‐methyltransferase inhibitors (Herman et al. 2023), and the putative protective effect of monoamine oxidase‐B inhibitors (Giladi, McDermott, Fahn, Przedborski, Jankovic, Stern, Tanner, and Parkinson Study Group 2001; Shoulson et al. 2002), in relation to FOG is both insufficient and inconsistent, precluding any evidence‐based recommendations for either their use or avoidance for the purpose of FOG prevention.

## 4 Pleiotropic Interventions as Potential Preventative Strategies for FOG

Although the drug development pipeline for PD is robust (McFarthing et al. 2024), no successful disease‐modifying therapies have been identified to date. Most trials have focused on pharmacological strategies targeting a single pathogenic mechanism, yielding largely disappointing results. A plausible explanation is that PD progression, and by extension the development of FOG, is driven by multiple interconnected pathophysiological mechanisms (this can be visualized as multiple ‘cogwheels’ that jointly drive the conveyor belt that symbolizes disease progression) (Bloem et al. 2025). Selectively targeting only one of these mechanisms (i.e., uncoupling just one cogwheel) may leave the other processes unchecked, allowing the remaining cogwheels to drive the conveyor belt forward (and allowing disease progression to continue). Consequently, effective prevention and treatment strategies for FOG perhaps require a multimodal or multifaceted approach, rather than relying on a singular intervention that tackles just one isolated disease mechanism. This concept is mirrored in other areas of medicine, such as tuberculosis, HIV, oncology and cardiovascular disease, where effective treatment often requires a ‘polypill’ targeting multiple pathways simultaneously. In this context, interventions that by virtue of their very nature are pleiotropic—or combinations of interventions that together modulate multiple disease‐relevant domains—may be particularly promising for reducing the risk or delaying the onset of FOG.

Lifestyle‐based interventions are inherently pleiotropic and may therefore hold promise for disease‐modification in PD and reducing the risk of developing FOG (Trinh et al. 2026). Below, we describe physical exercise, nutrition and stress management as the currently most extensively studied lifestyle domains in relation to FOG. Analogous to strategies employed in dementia prevention research (Ngandu et al. 2015; van Charante et al. 2016; Richard et al. 2019), a combination of the discussed lifestyle interventions targeting cardiovascular risk factors may also hold promise in the path towards prevention of FOG.

### 4.1 Physical Exercise

Among lifestyle‐based interventions, physical exercise is currently the most extensively studied and arguably the most advanced in terms of clinical translation. The SPARX trial, including 128 persons with ‘de novo’ (i.e., pharmacologically untreated) PD, demonstrated that 6 months of high‐intensity treadmill exercise, but not moderate‐intensity exercise, may slow motor deterioration compared to usual care (Schenkman et al. 2018). Similarly, the Park‐in‐Shape trial, including 130 persons with early stage PD, showed that 6 months of home‐based aerobic exercise using cycling on a stationary bicycle attenuated OFF‐state motor signs compared to stretching (van der Kolk et al. 2019). We acknowledge that these findings do not conclusively demonstrate disease modification, but they do cautiously offer some initial suggestions that sustained exercise could alter the trajectory of functional decline and thereby the risk of developing FOG in PD. Supporting this, a resting‐state functional and structural MRI study of an unselected subset of 56 participants from the Park‐in‐Shape trial showed that aerobic exercise, but not stretching, induced functional brain changes (Johansson et al. 2022). This included increased functional connectivity of the anterior putamen with the sensorimotor cortex relative to the posterior putamen, as well as increased functional connectivity in the right frontoparietal network, proportionally to fitness improvements. Furthermore, aerobic exercise reduced global brain atrophy and improved cognitive control (Johansson et al. 2022; Folkerts et al. 2024). Finally, although this remains speculative because of limitations in study design (i.e., the absence of a control group), some evidence suggests that exercise may increase striatal dopamine release, particularly in the anterior striatum (Sacheli et al. 2019). This could potentially facilitate compensatory gait control mechanisms and thereby contribute to delaying the onset of FOG.

Besides its effects on motor performance and cognitive control, exercise also targets several other established risk factors for FOG. Specifically, exercise supports overall cardiovascular health in the context of cerebral SVD, with beneficial effects on hypertension, hyperlipidemia, type 2 diabetes and obesity (Schootemeijer et al. 2020). Moreover, it improves sleep quality (Amara et al. 2020) and reduces symptoms of anxiety and depression (Costa et al. 2024). It is therefore now broadly recognized that physical activity and exercise programmes should be prescribed to all people with PD, obviously tailored to their physical and mental capacities (Tosserams et al. 2025).

### 4.2 Nutrition

Nutrition also shows potential in directly influencing the clinical outcomes for individuals with PD. Although exercise was studied extensively in the past years, the evidence for the benefits of nutrition is much more limited, as most studies on nutrition and PD were either observational or small pilot studies. Most nutritional interventions target the gut–brain axis through relieving gastrointestinal symptoms and addressing neuroinflammation.

Thus far, the majority of evidence relates to the relevance of the Mediterranean diet or the Mediterranean‐DASH Intervention for Neurodegenerative Delay (MIND) diet. An association between adherence to a Mediterranean diet and a lower risk of developing PD, as well as slower progression of PD, has been suggested (Bianchi et al. 2023). A higher adherence to these diets is inversely correlated with both motor and nonmotor symptoms in PD (Fox et al. 2022). Moreover, adherence to the Mediterranean diet is also linked to better cognitive function in people with PD (Paknahad et al. 2020). This cautiously suggests that nutrition might be used as a tool to target several relevant risk factors of FOG.

Another dietary pattern of interest is the ketogenic diet, which shows promising effects on motor symptoms through improved scores on the MDS‐UPDRS (de Farias et al. 2025), and that might also improve cognitive function (Krikorian et al. 2019). However, participants generally experience this dietary pattern as restrictive and difficult to adhere to in the long term, limiting the implementation of this strategy in clinical practice (Pokora et al. 2025). Related interventions, such as the use of nutritional supplements or probiotics and prebiotics, should also be mentioned in this context. Micronutrients deficiencies are common in PD and can contribute to symptom severity (Lister 2020). For example, B12 deficiency is related to postural instability and to decreases in motor and cognitive function in PD (Rekik et al. 2024). The use of prebiotic fibres can increase the level of short‐chain fatty acids, which in turn is correlated with improved motor and nonmotor function (Duan et al. 2024).

Taken together, the influence of nutrition on motor and nonmotor risk factors, as well as its role in cardiovascular health, suggests it could be used as a pleiotropic tool in the secondary prevention of FOG.

### 4.3 Stress Management

Stress management is another notable example of a pleiotropic approach that can address nonmotor risk factors of FOG such as anxiety or depression and that can reduce the risk of cardiovascular dysregulation due to chronic stress. Mind–body interventions, like mindfulness and yoga, are an important part of stress‐related interventions in PD. Especially mindfulness can have beneficial effects on stress (Goldberg et al. 2022) and can have synergetic effects when combined with exercise (Kwok et al. 2019). The evidence for yoga is more preliminary but also indicates a positive effect on motor symptoms (Suárez‐Iglesias et al. 2022). Currently, the ongoing MIND‐PD study is investigating whether Mindfulness‐Based Cognitive Therapy can affect motor symptoms in PD, including the exploration of brain imaging and cerebrospinal fluid biomarkers (van der Heide et al. 2024).

A new avenue for targeting stress in people with PD is art therapy (Gros et al. 2024). A recent uncontrolled pilot study of a 10‐week creative art therapy intervention in individuals with PD, demonstrated a significant reduction in anxiety, as well as an increase in well‐being and slight improvement in cognitive functioning (Spee et al. 2025). Similar results were later found in a controlled follow‐up study (Spee et al., Unpublished manuscript). Interestingly, healthcare consumption also went down in both studies, suggesting that in addition to health benefits, art therapy may also help in reducing the strain on an already overburdened health care system. These findings need to be confirmed in larger studies with longer follow‐up, including presence and severity of FOG as exploratory outcome.

## 5 Future Directions

The current evidence base for the secondary prevention of FOG is still quite limited, but it does provide a foundation for future work. The identified motor, nonmotor and vascular risk factors for FOG support the conceptualization of FOG as a multifactorial network disorder. Rather than acting in isolation, these factors likely interact and converge on a common final pathway, characterized by impaired integration across motor, cognitive and limbic networks. Many of the identified risk factors are at least partly modifiable through targeted or general interventions. Pleiotropic, primarily lifestyle‐based, interventions and combinations of complementary targeted interventions addressing specific risk factors across FOG‐associated motor, nonmotor and cardiovascular domains hold promise for the possible prevention of FOG and are more likely to be effective than single‐domain interventions. We propose that putative interventions aimed at preventing FOG should ideally be tested prior to its actual clinical onset, that is, in those individuals who have established PD, but who have not experienced actual FOG episodes yet. Further research is needed to determine the optimal timing of such preventative interventions and to determine whether tertiary prevention (i.e., limiting the severity of FOG among those who have already developed it) is also possible.

Although the rationale for the proposed approach is intuitive, it remains to be established whether the identified risk factors for FOG are truly causally involved in its development or rather represent epiphenomena of markers of overall disease progression. Consequently, it is uncertain whether targeting these specific risk factors will effectively prevent or delay the emergence of FOG or primarily modulate the associated symptoms.

To address these gaps, large‐scale, long‐term, longitudinal studies are warranted to better define the disease‐modifying potential of the various approaches and to elucidate the underlying biological mechanisms of any observed benefits. Future studies may broadly follow two complementary avenues: (i) investigating pleotropic interventions, aiming at prevention of overall disease progression, including prevention of development or worsening of specific risk factors for FOG, and (ii) investigating (a combination of) targeted training programmes directly aiming at the prevention of FOG. To this end, the currently ongoing Freezing of Gait–Clinical Outcomes Assessment (FOG‐COA) study aims to develop and validate a protocol to measure and evaluate FOG (NCT06519279). The protocol is reported in a companion paper in this special issue (Howe et al. 2026). The outcomes of this study will hopefully offer better insights into which outcome measures should be considered when studying the prevention of FOG both directly and indirectly, thereby strengthening future studies.

## Author Contributions

**Anouk Tosserams:** conceptualization, writing – original draft, visualization. **Jorik Nonnekes:** conceptualization, writing – review and editing. **Bastiaan R. Bloem:** conceptualization, writing – review and editing. **Elbrich M. Postma:** conceptualization, writing – original draft.

## Funding

This work was supported by ParkinsonNederland (PR0230).

## Conflicts of Interest

The authors declare no conflicts of interest.

## Data Availability

Data sharing is not applicable for the present contribution, as no original data were generated.
